# Ivermectin *vs* moxidectin for treating *Strongyloides stercoralis* infection: a systematic review

**DOI:** 10.1017/S0031182024001215

**Published:** 2024-11

**Authors:** Cesar Henriquez-Camacho, Jose A. Pérez-Molina, Dora Buonfrate, Paola Rodari, Eduardo Gotuzzo, Benilde Luengo, María Nieves Plana

**Affiliations:** 1 Faculty of Medicine, Universidad Francisco de Vitoria, Madrid, Spain; 2 Internal Medicine Unit, Hospital Universitario de Móstoles, Madrid, Spain; 3 National Reference Centre for Imported Tropical Diseases, Infectious Diseases Department, Hospital Universitario Ramón y Cajal, IRYCIS, Madrid, Spain; 4 CIBERINFEC, Instituto de Salud Carlos III, Madrid, Spain; 5 Department of Infectious Tropical Diseases and Microbiology, IRCCS Sacro Cuore Don Calabria Hospital, Negrar, Verona, Italy; 6 Faculty of Medicine, Universidad Peruana Cayetano Heredia, Lima, Peru; 7 Research Unit, Universidad Francisco de Vitoria (UFV), Madrid, Spain; 8 Health Technology Assessment Unit, Hospital Universitario Ramón y Cajal and Universidad de Alcalá (IRYCIS), CIBER Epidemiology and Public Health (CIBERESP), Madrid, Spain

**Keywords:** ivermectin, moxidectin, parasitological cure, *Strongyloides stercoralis*

## Abstract

The aim was to assess the efficacy of ivermectin *vs* moxidectin for treating *Strongyloides stercoralis* infection. Ovid MEDLINE, Embase and Web of Science databases were searched for studies comparing ivermectin and moxidectin from inception to February 2024. The outcomes: elimination of infection or parasitological cure, mortality and serious adverse events. We calculated odds ratios (ORs) with 95% confidence intervals (CIs) for dichotomous data. Heterogeneity was assessed using Chi2 test for statistical heterogeneity and results of the *I*^2^ statistic. Two trials met the inclusion criteria that included 821 adult participants. Both studies were conducted in southeast Asia (Cambodia and Laos). Neither trial included immunocompromised patients. The mean age of the participants ranged from 40 to 45 years old, with a similar distribution of males and females. For all participants, *S. stercoralis* infection was confirmed by Baermann method. The evidence was moderate for parasitological cure rate. Certainty was downgraded by 1 level because of imprecision. Moxidectin was not inferior to ivermectin: OR 0.67, 95% CI 0.36–1.25 (*P* = 0.21), *I*^2^ = 0%, 821 participants. No deaths were reported in either trial. One trial reported mild adverse events. In total, 153/726 (21%) participants had an adverse event. The most reported symptoms were abdominal pain and headache. There is evidence for moderate quality that moxidectin is non-inferior to, and as safe as ivermectin; however, more high-quality and well-designed trials are needed. For patients with some underlying immunosuppressive disorder, or in patients who are very young or very old, current data are insufficient to be recommended.

## Graphical abstract

**Figure:**
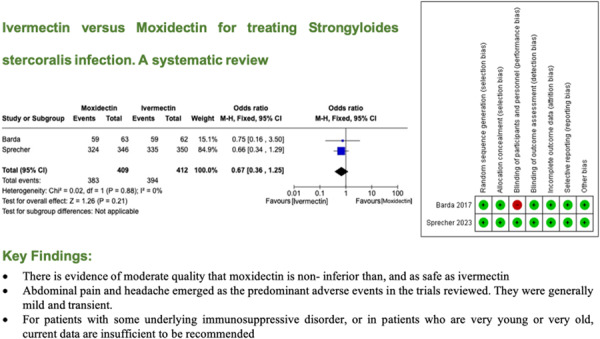

## Introduction

Strongyloidiasis is the infection caused by the intestinal parasitic worm *Strongyloides stercoralis*. This parasite is widely distributed worldwide, with a global prevalence of infection estimated at about 600 million cases in 2017 (Buonfrate *et al*., 2020). The highest burden is in southeast Asia, Africa and the Western Pacific region (Olsen *et al*., 2009; Buonfrate *et al*., 2020). Most infected individuals are asymptomatic, allowing the infection to remain undiagnosed and untreated for years. The highest risk of infection in the world is in rural areas where lack of access to sanitation and barefoot walking favour soil contamination by infective larvae (Buonfrate *et al*., 2023). Strongyloidiasis can be found in non-endemic areas due to increases in travel and migration from endemic to non-endemic countries (Bethony *et al*., 2006; Montes *et al*., 2010).

Three main clinical presentations of strongyloidiasis are acute infection, chronic infection, hyperinfection and dissemination. Chronic infection is characterized by low level of worm reproduction, and most infected people are asymptomatic. When symptoms are present, they are non-specific, allowing the infection to remain undiagnosed, thus untreated for years (Bisoffi *et al*., 2013). Of note, in immunosuppressed people strongyloidiasis can cause a serious and most often fatal illness: the hyperinfection syndrome. It describes an accelerated autoinfection, and the diagnosis implies the presence of signs and symptoms attributable to increased larval migration to organs beyond the range of the pulmonary auto-infective cycle (dissemination). The invasion of helminths into the mucosa is often associated with Gram-negative bacterial sepsis (Keiser and Nutman, 2004; Olsen *et al*., 2009). Delayed presentation, misdiagnosis and reduced access to clinical care impact mortality (Keiser and Nutman, 2004).

Conventional diagnosis is based on stool-based parasitological methods, such as direct-smear fecal microscopy, which has limited usefulness because of its extremely low sensitivity, Baermann sedimentation and agar plate culture. Several specimens should be collected on different days to improve the detection rate. The sensitivity of microscopic-based techniques might not be sufficient, especially in chronic infections where larval output is very low. However, the most sensitive techniques, the Baermann and agar plate methods, are too labour-intensive for use in an extensive population (Buonfrate *et al*., 2023). Serology assays are also available but sensitivity is low in very early infections and hyperinfection in immunosupressed patients. These include commercial enzyme-linked immunosorbent assays and other in-house techniques (Zaha *et al*., 2000; van Doorn *et al*., 2007; Bisoffi *et al*., 2014). Strongyloides DNA detection by real-time polymerase chain reaction is highly specific, with improved sensitivity compared to direct microscopy, but similar to that of Baermann and agar plate culture. As with other techniques, there is a lack of standardization of methods, including sample collection, preservation and DNA extraction methods (ten Hove *et al*., 2009; Gordon *et al*., 2024).

The Centers for Disease Control and Prevention and the World Health Organization (WHO) recommend ivermectin as the drug of choice for the treatment of strongyloidiasis, based on several clinical trials and meta-analysis (Henriquez-Camacho *et al*., 2016). Albendazole has lower efficacy than ivermectin, hence is considered a second-line drug (The Medical Letter, 2013).

The control of strongyloidiasis as a public health problem has been recently recommended by the WHO. However, the treatment is not universally available, although ivermectin is included in the WHO essential medicines list.

Massive use of ivermectin has been already carried out for decades, in the context of the elimination programmes for onchocerciasis and lymphatic filariasis. This has raised concerns about the possible emergence of drug resistance, that has been documented so far in veterinary but not in human medicine (Cobb and Boeckh, 2009). New drugs are needed to face this possible, but uncertain threat.

Moxidectin is a macrocyclic lactone that belongs to the same family of ivermectin and has been widely used by veterinarians (Cobb and Boeckh, 2009). In addition, moxidectin was used in humans in a randomized controlled trial (RCT) (NCT00790998) involving 1472 patients with onchocerciasis. The trial was conducted at 4 sites in West Africa (Opoku *et al*., 2018). Although moxidectin belongs to the same family of ivermectin, it has a different resistance pattern (Hofmann *et al*., 2022). This paves the way to also use it as an alternative treatment for strongyloidiasis.

The aim of this systematic review was to assess the efficacy of ivermectin *vs* moxidectin for treating *S. stercoralis* infection.

## Materials and methods

### Selection criteria

This systematic review was registered in the International prospective register of systematic reviews (PROSPERO) with the reference no. CRD42024494934, and it was conducted following the PRISMA 2020 statement criteria (Page *et al*., 2021). RCTs that evaluated the outcomes of moxidectin and ivermectin in *S. stercoralis* infection were included. The inclusion criteria were: participants aged 12 years and above; immunocompetent individuals; *S. stercoralis* infection confirmed by parasitological examination (at least 1 positive specimen). The following outcomes were considered: elimination of infection or parasitological cure, defined as any negative parasitological exam during follow-up period; mortality; serious adverse events, such as inpatient hospitalization or prolongation of existing hospitalization, persistent or significant disability/incapacity or life-threatening states. The search protocol followed the PRISMA guideline.

### Search strategy and study selection

We identified all relevant trials, regardless of their publication status (published, unpublished), using the search terms detailed in Appendix 1 in the following databases: Ovid MEDLINE (from January 1966 to February 2024), Embase (from January 1980 to February 2024) and Web of Science. The reference lists of the included studies were identified by the mentioned methods. Two reviewers (CH-C, PR) used Rayyan software to check the titles and abstracts of the literature independently, searching for potentially relevant articles (Ouzzani *et al*., 2016). We retrieved the full reports of potentially relevant trials and applied the inclusion criteria using an eligibility form. The third author (EG) resolved any disagreements. The eligible trials were scrutinized to exclude possible duplicates. In case of several publications reporting the same trial, the most recent was chosen. There were no restrictions based on language, sample size, age, gender, ethnicity or duration of follow-up.

### Data extraction and assessment of risk of bias

Two reviewers (DB and JAP-M) independently extracted data regarding the inclusion criteria, outcome data and adverse events. Any disagreement was resolved after evaluation by a third reviewer (EG). The risk of bias was assessed independently using a risk of bias Cochrane Collaboration form (Higgins *et al*., 2011), while the third reviewer resolved any disagreements. Generation of allocation sequence and allocation concealment was categorized as adequate, unclear or inadequate accordingly, using the criteria outlined in the Cochrane Handbook (Higgins *et al*., n.d.). We considered the following domains: random sequence generation (selection bias), allocation concealment (selection bias), blinding of participants and personnel (performance bias), blinding of outcome assessment (detection bias), incomplete outcome data (attrition bias), selective outcome reporting (reporting bias) and other sources of bias. We classified each domain as being at ‘low’, ‘high’ or ‘unclear’ risk of bias. A ‘Risk of bias’ graph was included in the analysis.

### Outcome assessment

The data extracted were dichotomous variables. We recorded the absolute number of events and participants in each group for all outcomes. Pooled odds ratios (ORs) with 95% confidence intervals (CIs) were reported. We calculated the proportion of loss to follow-up in each group. We reported data about methodological quality of trials, characteristics of participants, characteristics of interventions, characteristics of outcome measures, date of trial, trial authors, location of trial, sponsor of trial (specified, known or unknown), design (described as randomized or not), interventions (treatment, days, doses), and outcomes, mortality and serious adverse events. We calculated the proportion of loss to follow-up in each group.

### Statistical analysis

We calculated ORs with 95% CIs for dichotomous data. We planned to perform a pooled analysis by using a fixed or random effects model based on the level of heterogeneity. Heterogeneity was assessed using forest plots of study results to visually check for overlaps in CIs; Chi2 test for statistical heterogeneity (we considered trial results as heterogeneous with *P* < 0.10) and results of the *I*^2^ statistic. We judged the importance of the observed value of *I*^2^ according to the magnitude and direction of effects and the strength of evidence of heterogeneity (from 0 to 40% heterogeneity – might not consider important; from 30 to 60% heterogeneity – might be moderate; from 50 to 90% heterogeneity – might be substantial and from 75 to 100% heterogeneity – might be considerable) (Deeks *et al*., n.d.). We used the Review Manager 5 package, provided by Cochrane for data synthesis and analysis (Collaboration NCCTC., n.d.).

For the analysis of adverse events, the number of participants who experienced the adverse events were included. Data from trials that only reported the number of adverse events were not included, as it was possible that the same individual reported more than 1 adverse event.

We assessed the certainty of the evidence using the GRADE approach (Guyatt *et al*., 2011). Our confidence in the estimate of effect was evaluated in terms of study limitations, inconsistency of effect or unexplained heterogeneity, imprecision of results, indirectness and publication bias. We planned to evaluate the risk of publication bias graphically if more than 10 studies were included for the considered outcome. We presented a ‘Summary of findings’ table to summarize the key results of our review using the GRADEpro Guideline Development Tool.

## Results

**Figure 1.**
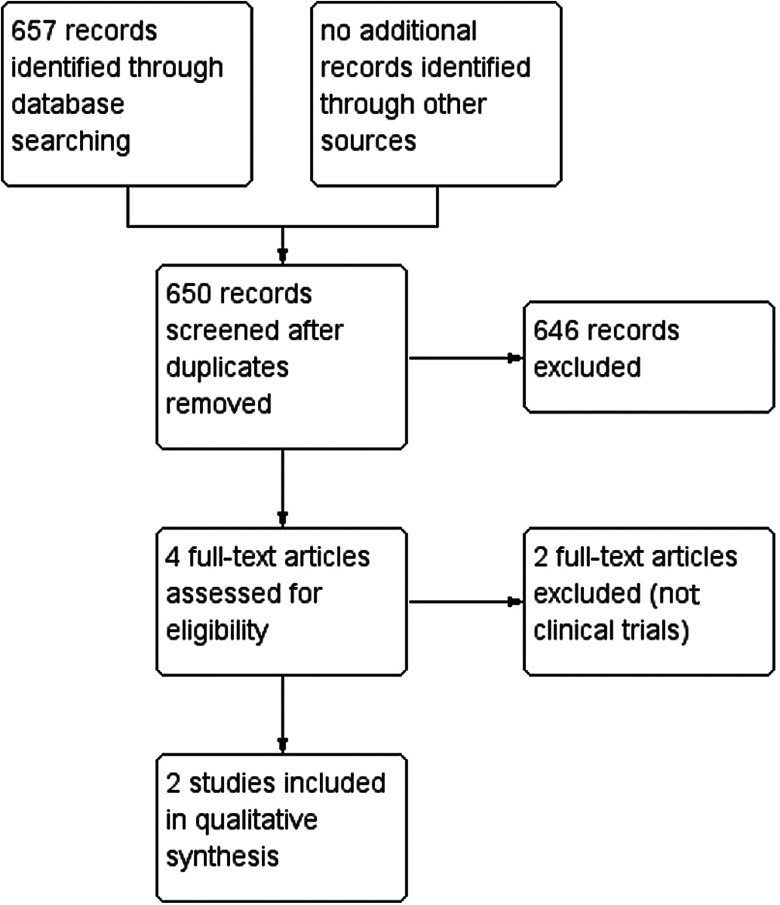
Study flow diagram.

The electronic search identified 296 references (PubMed), 247 references (Web of Science) and 114 references (Embase). A total of 650 records were identified after removing duplicates, 646 references were excluded based on title and abstract screening and 4 were included after full-text screening. Finally, 2 trials (Barda *et al*., 2017; Sprecher *et al*., 2024) met the inclusion criteria (Fig. 1). We excluded 2 trials because they were not RCTs (Hürlimann *et al*., 2023; Gandasegui *et al*., 2024).

Two RCTs, overall including 821 adult participants, were assessed in this review. Both studies were conducted in southeast Asia (Cambodia and Laos). None of the trials included immunocompromised patients. The mean age of the participants ranged from 40 to 45 years old, with a similar distribution of males and females. For all participants, *S.* s*tercoralis* infection was confirmed by the Baermann method. Pregnant and breastfeeding women were excluded, as well as women planning to become pregnant within 3 months after treatment. Participants with concomitant chronic diseases were also excluded in the Barda *et al*.'s (2017) trial (see Table 1).

**Table 1.** Characteristics of the included trials

Reference	Settings	Study period	Study design	Study population	Mean age in years (s.d.)	Female (%)
Barda *et al*. (2017)	Laos	April–June 2016	Randomized single-blinded, non-inferiority	127	MXD: 39.4 (12.9)	MXD: 52
					IVM: 40.7 (10.9)	IVM: 46
Sprecher *et al*. (2024)	Laos and central Cambodia	Laos:	Randomized, double-blind, parallel group, non-inferiority	696	Laos:	Laos:
		December 2020–February 2021			MXD: 45.4 (11.6)	MXD: 45
		Cambodia:			IVM: 44.8 (11)	IVM: 48
		February–May 2022			Cambodia:	Cambodia:
					MXD: 44.8 (12.8)	MXD: 48
					IVM: 44.9 (12.7)	IVM: 52

s.d., standard deviation.

### Interventions

In both trials, the dose of ivermectin was 200 μg kg^−1^ single dose (Stromectol^®^ from Merck Sharp & Dohme or IverP^®^ from Elea, Argentina), and the dose of moxidectin was fixed at 8 mg . In Sprecher *et al*. (2024), moxidectin formulation was 2 mg tablets (Development for Global Health; Melbourne, VIC, Australia), whereas in Barda *et al*. (2017) an oral suspension was used (Cydectin^®^ 0.1%; Zoetis, Switzerland). The analysis was per-protocol analysis. The follow-up period ranged from 14 to 25 days. The assessment of the outcome measures was based on the parasitological cure rate (see Table 2).

**Table 2.** Recruitment and following period of the included trials

Reference	Recruitment	Follow-up period (days)	Diagnostic method	Treatment	Mortality
Barda *et al*. (2017)	Ivermectin: 62 Moxidectin: 63	21–25	Baermann method	Ivermectin: 200 μg kg^−1^ sd	None
				Moxidectin: 8 mg sd	
Sprecher *et al*. (2024)	Laos:	14–21	Baermann method (2/6)	Ivermectin: 200 μg kg^−1^ sd	None
	MXD: 197			Moxidectin: 8 mg sd	
	IVM: 197				
	Cambodia:				
	MXD: 166				
	IVM: 166				

sd, single dose.

### Summary of findings

**Figure 2.**
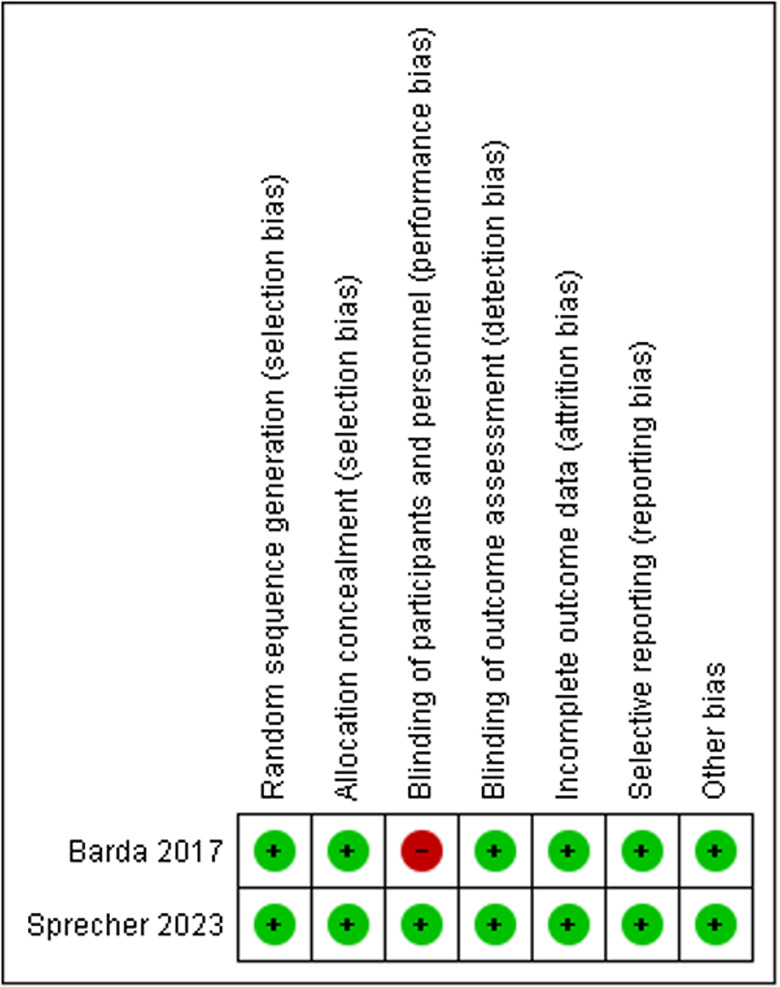
Risk of bias Summary.

The evidence was moderate for parasitological cure rate (see Appendix 2). Certainty was downgraded by 1 level because of imprecision (wide CIs). Regarding risk of bias, both RCTs reported adequate methods of allocation concealment and random sequence generation. Only 1 trial was double blinded for the participants and outcome assessment. Nevertheless, the lack of blinding may not have affected the results of the other study, because the primary outcome (parasitological cure and death) was objectively measured (see Fig. 2). We cannot evaluate the risk of publication bias due to the low number of studies.

### Effect of interventions

**Figure 3.**
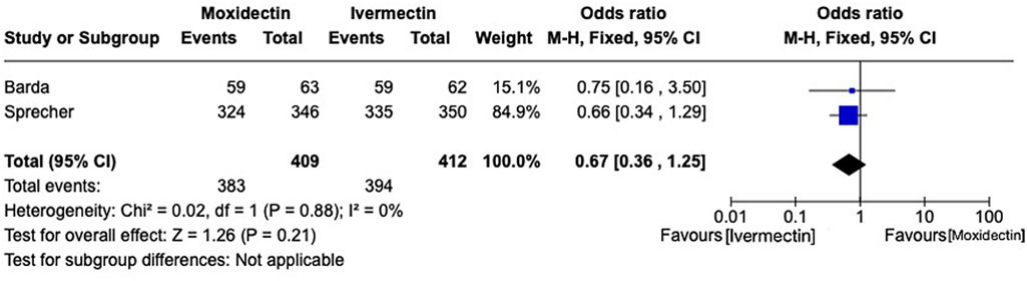
Comparison between moxidectin *vs* ivermectin for *Strongyloides stercoralis* infection. Outcome: parasitological cure rate.

The outcome was communicated in all reports. Both trials measured the parasitological cure at 14–21 days. Moxidectin was not inferior to ivermectin: OR 0.67, 95% CI 0.36–1.25 (*P* = 0.21), *I*^2^ = 0%, 821 participants (Fig. 3). No deaths were reported in any trial.

### Adverse events

One trial (Sprecher *et al*., 2024) reported mild adverse events that were unrelated to the study intervention. In total, 153/726 (21%) participants had an adverse event, which were equally distributed among the 2 treatment groups. The most reported symptoms were abdominal pain and headache. None of the patients stopped therapy or discontinued normal daily activities.

## Discussion

This systematic review aimed to summarize all the evidence from RCTs relating to the effectiveness of moxidectin compared to ivermectin in strongyloidiasis to provide current best evidence to base decisions for practice and further research.

The results suggest that there is evidence of moderate quality that moxidectin is non-inferior to, and as safe as ivermectin. We found no difference in the parasitological cure, although this result is based on only 2 trials with few patients.

Abdominal pain and headache emerged as the predominant adverse events in the trials reviewed. Given that ivermectin and moxidectin are members of the same pharmacological family, it is reasonable to hypothesize that they may exhibit similar adverse event profiles, despite potential differences in efficacy. The current review indicates that the adverse events associated with these drugs were generally mild and transient.

Both RCTs included immunocompetent participants over 11 years of age, with *S. stercoralis* infection detected by the Baermann method. Thus, we have no data comparing the efficacy of ivermectin or moxidectin on other clinical stages (acute strongyloidiasis or hyperinfection syndrome) because of the difficulty in finding patients in these stages or in severe cases of dissemination who agreed to undergo experimental treatment. Most patients have chronic infections. In the literature, there are case reports and case series describing the use of ivermectin for severe cases, but a robust estimate of its efficacy for these cases is not possible due to the lack of RCTs. There are no reports of moxidectin used in severe cases. Also, although evidence about the efficacy of ivermectin for treatment of strongyloidiasis in immunocompromised people is scarce, there are no data on the use of moxidectin in this vulnerable population.

Moreover, there are no data comparing the efficacy of the 2 drugs in children below 12 years of age, specifically because moxidectin is not registered for use in younger children. The ideal dose of ivermectin or moxidectin is unknown for very young or very old people as most of the trials did not include information about effectiveness in relation to age.

The effect of moxidectin or ivermectin in preventing new infections is not assessed. The trials included in this systematic review were not primarily designed to evaluate the effectiveness of moxidectin in preventing long-term new infections of strongyloidiasis and this outcome was not reported more than 25 days of following. Furthermore, moxidectin has a longer half-life in the plasma and a broader distribution based on its lipophilic properties (Hürlimann *et al*., 2023). This facilitates a longer-acting formulation, independent of the weight of the patient, and is widely used by veterinarians because of its long plasma half-life of 20–43 days (Prichard *et al*., 2012).

For Barda *et al*. (2017), we have considered that lack of blindness has a low risk of bias because the measurement of the outcome (parasitological cure) was done objectively. For both RCTs, there was insufficient information to assess the attrition bias of the trials included.

Publication bias is a major threat to the validity of systematic reviews. To minimize the risk of publication bias, we conducted a comprehensive search across numerous clinical trial databases. However, we consider it is unlikely to have occurred in this study because negative or non-conclusive RCTs are unlikely to remain unpublished.

We have identified a similar study, a Cochrane systematic review, published in 2011 comparing efficacy and safety of ivermectin and benzimidazoles for *S. stercoralis* infection (Henriquez-Camacho *et al*., 2016). The previous review did not include moxidectin, as this is drug has only recently been tested for this infection.

The main advantage of moxidectin over ivermectin is its weight-independent dosing, being an alternative treatment in patients with extreme low weight (mainly in poor settings) or obesity (in developed countries). This characteristic can be of interest in the context of mass drug administration, as it avoids the need of using scales or height poles. Ivermectin is currently employed for mass treatment in the context of the elimination programmes targeting onchocerciasis and lymphatic filariasis (Heukelbach *et al*., 2004). Indirect evidence from those programmes demonstrated a significant reduction in strongyloidiasis prevalence in endemic communities (Traore *et al*., 2012), posing the basis for the use of this drug for public health control of *S. stercoralis* infection (Stroffolini *et al*., 2023). Based on the results of clinical trials it is likely moxidectin will be similarly approved in the future for strongyloidiasis.

The main limitation of this study is the low number of RCTs, which did not permit us to carry out a meta-analysis. The overall evidence was deemed of good quality; however the study by Sprecher presented some discrepant data between study sites that would deserve further evaluation, at least on efficacy of moxidectin in different settings and populations.

## Data Availability

Dataset used and/or analysed during the current study is available upon reasonable request to the corresponding author.

## Appendix 1. Search strategy

**Table:**

Medline	Web of Science	Embase
#1 Strongyloidea OR Strongyloidiasis OR strongyloid* OR Strongylida Infections OR Strongyloides stercoralis OR Strongyloides #2 controlled clinical trial OR randomized controlled trial #3 randomized or randomised #4 placebo #5 dt.fs. #6 randomly OR trial OR groups #7 2 OR 3 OR 4 OR 5 OR 6 #8 exp animals not humans #9 7 not 8 #10 1 AND 9 #11 Ivermectin #12 Moxidectin #13 Anthelmintics OR moxidectin #14 11 OR 12 OR 13 #15 10 AND 14	#1 strongyloid* #2 ivermectin or moxidectin #3 1 AND 2 #4 randomized controlled trial OR random* OR control* OR group* OR cluster* OR placebo* OR trial* OR assign* OR allocat* OR prospectiv* OR random* OR control* OR group* OR cluster* OR placebo* OR trial* OR assign* OR allocat* OR prospectiv* #5 3 AND 4	#1 strongyloides OR strongyloides stercoralis OR strongyloidiasis #2 ivermectin #3 moxidectin #4 anthelmintic agent OR antiparasitic agent #5 2 OR 3 OR 4 #6 1 AND 5 #7 random* OR factorial* OR placebo* OR assign* OR allocat* OR crossover* #8 (blind* or mask*) AND (single OR double OR triple or treble) #9 crossover procedure #10 double blind procedure OR single blind procedure #11 randomization OR placebo #12 parallel design OR latin square design #13 randomized controlled trial #14 7 OR 8 OR 9 OR 10 OR 11 OR 12 OR 13 #15 animal OR nonhuman OR animal experiment OR animal model #16 human #17 15 not 16 #18 14 not 17 #19 6 AND 18
296 references	247 references	114 references

## Appendix 2. Summary of findings for the main comparison: moxidectin *vs* ivermectin: cure rate

**Table:**

Certainty assessment							No. of patients		Effect		Certainty	Importance
No. of studies	Study design	Risk of bias	Inconsistency	Indirectness	Imprecision	Other considerations	Moxidectin	Ivermectin	Relative (95% CI)	Absolute (95% CI)		
2	Randomized trials	Not serious	Not serious	Not serious	Serious	None	383/409 (93.6%)	394/412 (95.6%)	OR 0.67 (0.36–1.25)	20 fewer per 1000 (from 69 fewer to 8 more)	Moderate^a^ ⊕⊕⊕◯	Critical

CI, confidence interval; OR, odds ratio.

a Downgraded 1 level due to imprecision.

*GRADE Working Group grades of evidence*.

*High-certainty:* Further research is very unlikely to change our confidence in the estimate of effect.

*Moderate-certainty:* Further research is likely to have an important impact on our confidence in the estimate of effect and may change the estimate.

*Low-certainty:* Further research is very likely to have an important impact on our confidence in the estimate of effect and is likely to change the estimate.

*Very low-certainty:* We are very uncertain about the estimate.
